# Large-scale molecular survey for piroplasmids in Iberian wild carnivores

**DOI:** 10.1007/s00436-024-08425-5

**Published:** 2024-12-13

**Authors:** Javier Millán, Rocío Checa, Álvaro Oleaga, Alejandro Rodríguez, Nieves Negre, Luis Llaneza, Roser Velarde, Guadalupe Miró

**Affiliations:** 1 Instituto Agroalimentario de Aragón-IA2 (Universidad de Zaragoza-CITA), 50013 Saragossa, Spain; 2 Fundación ARAID, Avda. Ranillas 1, 50018 Saragossa, Spain; 3 Facultad de Ciencias de la Vida, Universidad Andres Bello, Santiago, Chile; 4 Microbiology and Parasitology Department, Faculty of Pharmacy, Universidad Complutense of Madrid, Madrid, Spain; 5 SERPA, Sociedad de Servicios del Principado de Asturias S.A., 33203 Gijón, Asturias Spain; 6 Department of Conservation Biology and Global Change, Estación Biológica de Doñana, CSIC, Américo Vespucio 26, 41092 Seville, Spain; 7 Fundació Natura Parc, Santa Eugènia, 07142 Balearic Islands, Spain; 8 A.RE.NA. Asesores en Recursos Naturales SL, Perpetuo Socorro 12-Entresuelo 2B, 27003 Lugo, Spain; 9 Facultade de Ciencias, Universidade da Coruña, Campus da Zapateira S/N, 15008 A Coruña, Spain; 10 Department Medicina I Cirurgia Animals, Facultat de Veterinària, Universitat Autònoma de Barcelona, Barcelona, Spain; 11 Animal Health Department, Faculty of Veterinary Medicine, Universidad Complutense of Madrid, Madrid, Spain

**Keywords:** Canidae, Felidae, Mustelidae, Piroplasmida, Viverridae

## Abstract

Piroplasmids are vector-borne hemoprotozoan parasites belonging to the phylum Apicomplexa that are of veterinary and medical importance. Wild carnivores are hosts for diverse piroplasmids, some of which are highly pathogenic for domestic dogs and cats. A large-scale survey including samples from 244 individuals belonging to eleven different species that were opportunistically obtained between 1993 and 2015 in four Autonomous Regions in Spain were tested for piroplasmid DNA with two different nested-PCR assays targeting the 18S rRNA gene. Sixty of 85 Eurasian badgers (*Meles meles*), 11 of 42 red foxes (*Vulpes vulpes*), and 1 of 10 stone martens (*Martes foina*) resulted positive. In contrast, 46 wolves (*Canis lupus*), 26 genets (*Genetta genetta*), 22 pine martens (*Martes martes*), and other less-represented species were negative. Sequencing revealed that all foxes and one badger were parasitized by *Babesia vulpes*, and the remaining badgers and the stone marten by *Babesia* sp. badger type A (BBTA). The prevalence of BBTA in Catalonian badgers was significantly lower in Alpine than in Continental and Mediterranean climates. This study confirms that badgers and ref foxes constitute the natural hosts of BBTA and *B. vulpes*, respectively, with occasional spillovers to other species.

**Supplementary Information:**

The online version contains supplementary material available at 10.1007/s00436-024-08425-5.

## Introduction

Piroplasmids are a group of vector-borne hemoprotozoan parasites belonging to the phylum Apicomplexa that have importance from the veterinary and medical point of view (Mehlhorn and Schein 1984). Piroplasmids are the second most common parasites in the blood of mammals after trypanosomes (Schnittger et al. 2012). Wild carnivores are well-known hosts for piroplasmids (Alvarado-Rybak et al. 2016). In many cases, wild carnivores are considered natural hosts for a given species, such as different wild felines for *Cytauxzoon* spp. (Reichard et al. 2005; Birkenheuer et al. 2008; Panait et al. 2021), the red fox (*Vulpes vulpes*) for *Babesia vulpes* (Baneth et al. 2015, 2019, Otranto et al. 2015), and some African carnivores such as the black-backed jackal (*Canis mesomelas*) for *Babesia rossi* (Penzhorn 2011). These parasite species are, in general, very pathogenic to domestic cats and dogs (Lloret et al. 2015; Miró et al. 2015; Schnittger et al. 2022).

In the Iberian Peninsula, diverse piroplasmids have been detected in the Carnivora, including *B. vulpes* in the red fox (Giménez et al. 2009, Cardoso et al. 2013, Barandika et al. 2016, Millán et al. 2016b, Checa et al. 2018, Ortuño et al. 2022), the so-called *Babesia* sp. badger type A (BBTA) and the *Babesia* sp. badger type B (BBTB) in the Eurasian badger (*Meles meles*) (Barandika et al. 2016, Ortuño et al. 2022), another *Babesia* genotype belonging to the *microti* group in a badger (Giménez et al. 2009), *Babesia vogeli* in a stone marten (*Martes foina*) (Millán et al. 2016b), a *Babesia* species closely related to a sequence from red foxes in Israel in a European wildcat (*Felis silvestris*) (Ortuño et al. 2022), and *Cytauxzoon* spp. in Iberian lynx (*Lynx pardinus*) and wildcat (Luaces et al. 2005, Millán et al. 2007, Meli et al. 2009, Barandika et al. 2016, León et al. 2017, Ortuño et al. 2022).

Wild carnivores are pivotal in the epidemiology of piroplasmids (Otranto et al. 2015, Alvarado-Rybak et al. 2016). In consequence, this study aimed to provide an update on the knowledge of the identity, prevalence, and distribution of piroplasmids in Iberian wild carnivores from diverse areas across Spain.

## Material and methods

### Animal sampling

The sample consisted of 244 individuals belonging to eleven different species, opportunistically obtained between 1993 and 2015 in four Autonomous Regions in Spain: Catalonia, Galicia, Asturias, and Balearic Islands (Table 1). Carnivores sampled in Catalonia and Mallorca (Balearic Islands) were road-killed individuals collected during official regional monitoring programs (see details in Calatayud et al. 2020). The carnivores sampled from Catalonia in this survey were individuals who differed from those included in the study by Millán et al. (2016b). Wolves were sampled either alive or dead in Asturias and Galicia (see details in Millán et al. 2016a). A piece of spleen was collected from animals sampled post-mortem; whole blood was collected from wild-caught wolves and stored in EDTA tubes. Samples were frozen at − 20 °C until analysis.

**Table 1:** Number of samples for each carnivore species in four Spanish regions. The number of positives to piroplasmid infection, as revealed by primers BT1/2 F/R and BTFox1 F/R, are indicated in parentheses. Prevalence and confidence intervals are only calculated for species with a sample size larger than 20

Family/species	Asturias	Catalonia	Galicia	Mallorca	Total tested	Prevalence	95% Confidence intervals
Family Canidae							
Wolf (*Canis lupus*)	20		22		42	0%	0.0–8.4
Red fox (*Vulpes vulpes*)		42 (11)			42 (11)	26.1%	13.8–42.0
Family Mustelidae							
Eurasian badger (*Meles meles*)		85 (60)			85 (60)	70.6%	50.7–80.0
Pine marten (*Martes martes*)		1		21	22	0%	0.0–15.4
Stone marten (*Martes foina*)		10 (1)			10 (1)		
European mink (*Mustela lutreola*)		1			1		
Least weasel (*Mustela nivalis*)		2		2	4		
American mink (*Neogale vison*)		2			2		
Family Procyonidae							
South American coati (*Nasua nasua*)				1	1		
Family Felidae							
European wildcat (*Felis s. silvestris*)		2			2		
Family Viverridae							
Common genet (*Genetta genetta*)		26		7	33	0%	0.0–10.1

### DNA extraction and molecular diagnosis

DNA extraction of homogenized samples was performed with a pressure filtration method (QuickGene® DNA tissue kit S, Fujifilm Life Science, Tokyo, Japan). DNA samples were screened for piroplasm detection using two nested PCR assays targeting the 18S RNA gene. Initially, a nested-PCR assay was performed using the primers BT1 F/R followed by BT2 F/R (Thermo Fisher Scientific, USA), with slight modifications to the protocol outlined by Jefferies et al. (2007). Briefly, for the first amplification, 2 µl of DNA was added to 23 µl of master mix containing 0.5 µM of each primer (BT1 F/R), 200 µM (each) deoxyribonucleotides (dATP, dTTP, dGTP, dCTP), 1.5 mM MgCl_2_, 2.5 µl 10 × PCR buffer, and 0.75 units of Polymerase (5 U/µl) (Biotools B&M Labs S.A., Spain). The thermocycling program (thermocycler GenAmp® PCR System 2720, Applied Biosystem, Spain) was as follows: an initial cycle at 94 °C for 3 min, 58 °C for 1 min, and 72 °C for 2 min; followed by 45 cycles of 94 °C for 30 s, 58 °C for 20 s, and 72 °C for 30 s, with a final extension at 72 °C for 7 min. The conditions for the second PCR reaction were the same as in the first one, except that 2 µl of the amplified product (obtained from the first amplification round) was added to 23 µl of master mix containing the primer set BT2 F/R and the annealing temperature was 62 °C. Sample sizes ranging from 800 to 850 bp were taken as positive for the DNA of Piroplasmida (*Babesia*, *Theileria,* or *Cytauxzoon*).

A second nested PCR method, which is highly sensitive for the specific detection of *Babesia vulpes*, was performed. The methodology employed was adapted from that previously described by Bartley et al. (2016). For the first amplification round, 2 µl of the DNA was added to a final volume of 23 µl master mix containing 0.2 µM of each primer BT1F and BTH-R (Criado-Fornelio et al. 2003), 2.5 µl of 10 × PCR buffer, 0.5 µl of dNTP Mix (10 mM each), and 0.75 units of Tth Plus DNA polymerase (5 U/µl) (Biotools B&M Labs S.A., Spain). The thermocycling program used in the first amplification was as follows: an initial cycle at 80 °C for 2 min, followed by 94 °C for 5 min, then 35 cycles at 94 °C for 1 min, 55 °C for 1 min, and 72 °C for 1 min, with a final extension step at 72 °C for 5 min. The conditions for the second amplification reaction were identical to the first one, except that it contained 2 µl of the product obtained after the first amplification round and 0.2 µM of each primer BTFox1 F/R (Bartley et al. 2016). In addition, an annealing temperature of 52 °C for 1 min was used. Positive DNA samples for *Babesia* spp. resulted in a final fragment size of around 655 bp.

Amplified PCR products (10–15 µl) were examined by electrophoresis on a 1.5% agarose gel stained with SYBR® Safe Gel Stain (1:10,000) (Thermo Fisher Scientific, USA) and visualized under UV light using a transilluminator (Clare Chemical, USA). A sample was regarded as positive if an amplicon of the expected length (according to the corresponding PCR assay) was present in a duplicate reaction. Negative and positive controls (*B. canis* and *B. vulpes* DNA) were included in each PCR reaction. Positive samples were sent to the Genomics Unit for purification and sequencing (Universidad Complutense de Madrid). The obtained sequences were analyzed using the software program Chromas v.2.6.6 and compared with sequences available in GenBank^©^ using the BLAST software (https://blast.ncbi.nlm.nih.gov/Blast.cgi).

### Statistical analysis

Differences among species, sex, and age groups were tested using Fisher’s exact test. Georeferenced badgers from Catalonia were assigned to one of the following climatic regions: Continental, Mediterranean, or Alpine. Differences in the prevalence of BBTA were compared between these regions also using Fisher’s exact test. Unfortunately, most of the information about the origin of foxes was lost, preventing us from reporting the geographical sites at which *B. vulpes* isolates had been sampled.

## Results

The “BTFox1” protocol resulted in 60 of 85 isolates sampled from badgers (70.6%, 95% confidence interval = 50.7–80.0), 11 of 42 isolates sampled from red foxes (26.1%, 13.8–42.0), and 1 of 10 isolates sampled from stone martens (10.0%, 0.02–40.5) positive for piroplasmid DNA. The protocol “Babesia BT1/2” generated 17, three, and one piroplasmid DNA positive results for badger, fox, and stone marten, respectively. All individuals from the remaining species tested were negative for piroplasmid DNA. Prevalence was higher in badger than in all the other species with a sample size > 20 (in all cases, Fisher’s *p* < 0.0001). Prevalence in fox was higher than in wolf, genet, and pine marten (in all cases, Fisher’s *p* < 0.01). No sex or age-related differences were found for badgers or foxes (in all cases, Fisher’s *p* > 0.05). The prevalence of BBTA in badgers was lower in the Alpine (63%, *n* = 27) than in the Continental (100%, *n* = 18; Fisher’s *p* = 0.003) and the Mediterranean (91%, *n* = 22; Fisher’s *p* = 0.04) climates.

Sequencing indicated that all foxes (*n* = 10 readable sequences) and one badger were parasitized by *B. vulpes*, whereas the remaining badgers (*n* = 33 sequences) and the stone marten were infected by BBTA. All the sequences showed between 99.8 and 100% identity with one BBTA isolate previously reported in badgers from Basque Country (GenBank: KT223484.1) or *B. vulpes* isolates identified both in red foxes and dogs from north and north-western Spain (GenBank: MK591948.1, KT223483.1, AY534602.1) or red foxes from European countries (GenBank: MW295528, KT580785.1). The BBTA sequence from the stone marten and *B. vulpes* from the badger were deposited in the GenBank database under accession numbers PP979490 and PP979536, respectively. In addition, representative consensus sequences of *B. vulpes* from red foxes and BBTA from badgers were deposited under the accession numbers PP980987 and PP979585.

## Discussion

This is the most extensive survey of piroplasmids in wildlife, both in terms of geography and diversity of species tested, conducted to date in the Iberian Peninsula, and one of the largest in wild carnivores worldwide. The prevalence of piroplasmid DNA in the studied badger population is almost identical to the one previously observed in the UK (Bartley et al. 2017). However, Bartley et al. (2017) observed a higher prevalence in blood than spleen samples. Thus, since only badger spleen samples were used in the present survey, the actual prevalence in our badger population could have been underestimated. In Spain, no large sample sizes of badgers were gathered before, but previous studies found 0/3, 1/5, and 14/24 positive individuals in NW, N, and S Spain, respectively (Giménez et al. 2009, Millán et al. 2016b, Ortuño et al. 2022). Elsewhere in Europe, occurrences also tended to be high (up to 91% in Italy; Battisti et al. 2020). Reasons for this high prevalence in this mustelid, when compared to other related species, might be related to its social behavior, which may facilitate transmission, or its strictly terrestrial lifestyle, which may expose badgers to a higher risk to be infested by ticks. In contrast, arboreal species such as martens or genets that live solitary should have a lower risk to be infested by ticks. However, this would not explain why wolves sampled in the present study, which are also social and terrestrial species, were not found to be parasitized by piroplasmids. Previous surveys of European wolves showed prevalences of *Babesia* spp. of 20% in Croatia (Beck et al. 2017) and 7% and 40% in Southern and Northern Italy, respectively (Santoro et al. 2019; Battisti et al. 2020). In the latter study, prevalence in badgers was also significantly higher than in wolves, suggesting that differences in susceptibility and/or exposure do exist between these species.

All readable sequences but one retrieved from badgers corresponded with the so-called BBTA. This piroplasmid was first described in European badgers from the Basque Country in Northern Spain (Barandika et al., 2016) and later found elsewhere in Europe (Bartley et al. 2017, Santoro et al. 2019, Guardone et al. 2020, Ortuño et al. 2022). Very closely related sequences have been detected in Asian badgers (*Meles leucurus*; Sang et al. 2021). This piroplasmid has been also found in hunting dogs in Hungary (Hórnok et al. 2018). Thus, our study agrees with previous reports that suggest that the Eurasian badgers are the natural host for BBTA. However, the other badger-associated piroplasmid, BBTB, appears to be absent in the studied animal population of Catalonia. Badgers infected with BBTA did not show clinical signs of disease, but it was reported to be pathogenic to dogs (Hórnok et al. 2018). This finding shows a parallel to *Babesia vulpes*, which does not appear to be pathogenic for foxes, but is pathogenic for dogs (Miró et al. 2015).

The vector for BBTA is so far unknown. Hórnok et al. (2018) proposed *Ixodes canisuga* as a vector of BBTA since they found DNA of BBTA in 18 out of 27 of *I. canisuga* specimens retrieved from hosts but in none of six *I. hexagonus* specimens. On the other hand, BBTA was identified in two *I. ricinus* of which one was collected from red deer and the other from roe deer (del Cerro et al. 2022). However, none of these studies presented experimental evidence for vector competence of this tick species for BBTA transmission (see Estrada-Peña et al. 2021). Our geographical analyses showed that BBTA prevalence among badgers from the Alpine climatic region (i.e., the Pyrenees) was lower than that in milder climates. We assume that cold weather in this area may be detrimental to the development and survival for the potential BBTA tick vector.

We report for the first time the presence of BBTA in a stone marten. Previously, *B. vogeli* was identified in this species in Catalonia (Millán et al. 2016b), confirming that stone martens, as an additional mustelid besides the badger, are also susceptible to this *Babesia* variant. This study confirms the circulation of *B. vulpes* in Catalonia as previously reported (Millán et al. 2016b). *Babesia vulpes* is important in the veterinary practice as it was shown to be highly pathogenic for dogs causing a severe disease refractory to the treatment with conventional babesicides (Miró et al. 2015). The prevalence of *B. vulpes* in the red fox sample was reported to be much lower in Catalonia than in Galicia in NE Spain (Checa et al. 2018); however, prevalence values widely vary between studies from different areas in Europe (Najm et al. 2014, Farkas et al. 2015, Koneval et al. 2017, Battisti et al. 2020). Estimation of prevalence is influenced by factors such as the age of the host, the season of sampling, the type of sample, and the molecular detection protocol. Remarkably, one badger was found positive for *B. vulpes*, which may represent an additional host besides the fox but also be an example of a spillover infection.

In conclusion, our survey advances the current knowledge on the distribution and identity of piroplasmids in previously unexplored species and regions of the Iberian Peninsula. Furthermore, our results suggest that BBTA represents a possible new *Babesia* species that awaits description.

## Supplementary Information

Below is the link to the electronic supplementary material.

Supplementary file1 (XLSX 11151 KB)

## Data Availability

All data supporting the findings of this study are available within the paper and its Supplementary Information.

## References

[CR1] Alvarado-Rybak M, Solano-Gallego L, Millán J (2016) A review of piroplasmid infections in wild carnivores worldwide:importance for domestic animal health and wildlife conservation. Parasit Vectors 9(1):538. 10.1186/s13071-016-1808-7 27724937 10.1186/s13071-016-1808-7 PMC5057422

[CR2] Baneth G, Florin-Christensen M, Cardoso L, Schnittger L (2015) Reclassification of *Theileria annae* as *Babesia vulpes* sp. nov. Parasit Vectors 8(1):207 25890372 10.1186/s13071-015-0830-5 PMC4393874

[CR3] Baneth G, Cardoso L, Brilhante-Simões P, Schnittger L (2019) Establishment of *Babesia vulpes* n. sp. (Apicomplexa: Babesiidae), a piroplasmid species pathogenic for domestic dogs. Parasit Vectors 12(1):129. 10.1186/s13071-019-3385-z 10.1186/s13071-019-3385-z PMC6434798 30909951

[CR4] Barandika JF, Espí A, Oporto B, Del Cerro A, Barral M, Povedano I, García-Pérez AL, Hurtado A (2016) Occurrence and genetic diversity of piroplasms and other apicomplexa in wild carnivores. Parasitol Open 2:e6 10.1016/j.ttbdis.2015.10.019 26596894

[CR5] Bartley PM, Hamilton C, Wilson C, Innes EA, Katzer F (2016) Detection of *Babesia annae* DNA in lung exudate samples from red foxes (*Vulpes vulpes*) in Great Britain. Parasit Vectors 9:84. 10.1186/s13071-016-1364-1 26867572 10.1186/s13071-016-1364-1 PMC4751633

[CR6] Bartley PM, Wilson C, Innes EA, Katzer F (2017) Detection of Babesia DNA in blood and spleen samples from Eurasian badgers (*Meles meles*) in Scotland. Parasitology 144(9):1203–1210 28696186 10.1017/S0031182017000476

[CR7] Battisti E, Zanet S, Khalili S, Trisciuoglio A, Hertel B, Ferroglio E (2020) Molecular survey on vector-borne pathogens in alpine wild carnivorans. Front Vet Sci 7:1. 10.3389/fvets.2020.00001 32039255 10.3389/fvets.2020.00001 PMC6989405

[CR8] Beck A, Huber D, Polkinghorne A, Kurilj AG, Benko V, Mrljak V, Reljić S, Kusak J, Reil I, Beck R (2017) The prevalence and impact of *Babesia canis* and *Theileria *sp. in free-ranging grey wolf (*Canis lupus*) populations in Croatia. Parasites & vectors 10:1–9 28376903 10.1186/s13071-017-2106-8 PMC5379697

[CR9] Birkenheuer A, Marr H, Warren C, Acton A, Mucker E, Humphreys J, Tucker M (2008) *Cytauxzoon felis* infections are present in bobcats (Lynx rufus) in a region where cytauxzoonosis is not recognized in domestic cats. Vet Parasitol 153:126–130 18295403 10.1016/j.vetpar.2008.01.020

[CR10] Calatayud O, Esperón F, Velarde R, Oleaga Á, Llaneza L, Ribas A, Negre N, de La Torre A, Rodríguez A, Millán J (2020) Genetic characterization of Carnivore Parvoviruses in Spanish wildlife reveals domestic dog and cat-related sequences. Transbound Emerg Dis 67(2):626–634 31581349 10.1111/tbed.13378

[CR11] Cardoso L, Cortes HC, Reis A, Rodrigues P, Simões M, Lopes AP, Vila-Viçosa MJ, Talmi-Frank D, Eyal O, Solano-Gallego L, Baneth G (2013) Prevalence of *Babesia microti*-like infection in red foxes (*Vulpes vulpes*) from Portugal. Vet Parasitol 196(1–2):90–95 23352108 10.1016/j.vetpar.2012.12.060

[CR12] Checa R, López-Beceiro AM, Montoya A, Barrera JP, Ortega N, Gálvez R, ... Miró G (2018) *Babesia microti*-like piroplasm (syn. *Babesia vulpes*) infection in red foxes (*Vulpes vulpes*) in NW Spain (Galicia) and its relationship with *Ixodes hexagonus*. Veterinary parasitology, 252, 22–28 10.1016/j.vetpar.2018.01.011 29559146

[CR13] Criado-Fornelio A, Martinez-Marcos A, Buling-Saraña A, Barba-Carretero JC (2003) Molecular studies on Babesia, *Theileria* and *Hepatozoon* in Southern Europe. Part II. Phylogenetic analysis and evolutionary history. Vet Parasitol 114:173–194 12788253 10.1016/s0304-4017(03)00141-9

[CR14] Del Cerro A, Oleaga A, Somoano A, Barandika JF, García-Pérez AL, Espí A (2022) Molecular identification of tick-borne pathogens (*Rickettsia spp., Anaplasma phagocytophilum, Borrelia burgdorferi* sensu lato, *Coxiella burnetii* and piroplasms) in questing and feeding hard ticks from North-Western Spain. Ticks Tick-borne Dis 13(4):101961 35490548 10.1016/j.ttbdis.2022.101961

[CR15] Estrada-Peña A, Cevidanes A, Sprong H, Millán J (2021) Pitfalls in tick and tick-borne pathogens research, some recommendations and a call for data sharing. Pathogens 10(6):712 34200175 10.3390/pathogens10060712 PMC8229135

[CR16] Farkas R, Takács N, Hornyák Á, Nachum-Biala Y, Hornok S, Baneth G (2015) First report on *Babesia cf. microti* infection of red foxes (*Vulpes vulpes*) from Hungary. Parasites Vectors 8:4–9 25623386 10.1186/s13071-015-0660-5 PMC4316759

[CR17] Gimenez C, Casado N, Criado-Fornelio A, de Miguel FA, Dominguez-Peñafiel G (2009) A molecular survey of Piroplasmiand Hepatozoon isolated from domestic and wild animals in Burgos (northern Spain). Vet Parasitol 162(1–2):147–50. 10.1016/j.vetpar.2009.02.021 19297099 10.1016/j.vetpar.2009.02.021

[CR18] Guardone L, Ebani VV, Verin R, Nardoni S, Consolazione A, Bennett M, Mancianti F (2020) Molecular detection of arthropod-borne pathogens in Eurasian badgers (*Meles meles*) from the United Kingdom. Animals 10(3):446 32155963 10.3390/ani10030446 PMC7143893

[CR19] Hórnok S, Horváth G, Takács N, Kontschán J, Szőke K, Farkas R (2018) Molecular identification of badger-associated *Babesia* sp. DNA in dogs: updated phylogeny of piroplasms infecting Caniformia. Parasites Vectors 11(1):1–6 29642942 10.1186/s13071-018-2794-8 PMC5896074

[CR20] Jefferies R, Ryan UM, Irwin PJ (2007) PCR-RFLP for the detection and differentiation of the canine piroplasm species and its use with filter paper-based technologies. Vet Parasitol 144:20–27. 10.1016/j.vetpar.2006.09.022 17127005 10.1016/j.vetpar.2006.09.022

[CR21] Koneval M, Miterpáková M, Hurníková Z, Blanarová L, Víchová B (2017) Neglected intravascular pathogens, *Babesia vulpes* and haemotropic *Mycoplasma* spp. in European red fox (Vulpes vulpes) population. Vet Parasitol 243:176–182 28807289 10.1016/j.vetpar.2017.06.029

[CR22] León CI, García-Bocanegra I, McCain E, Rodríguez E, Zorrilla I, Gómez AM, ... Gómez-Guillamón F (2017) Prevalence of selected pathogens in small carnivores in reintroduction areas of the Iberian lynx (*Lynx pardinus*). The Veterinary Record, 180(10), 252 10.1136/vr.104038 28062843

[CR23] Lloret A, Addie DD, Boucraut-Baralon C, Egberink H, Frymus T, Gruffydd-Jones T, ... European Advisory Board on Cat Diseases (2015) Cytauxzoonosis in cats: ABCD guidelines on prevention and management. J Feline Med Surg, 17(7), 637–641 10.1177/1098612X15589878 PMC11148930 26101317

[CR24] Luaces I, Aguirre E, García-Montijano M, Velarde J, Tesouro M, Sánchez C et al (2005) First report of an intraerythrocytic small piroplasm in wild Iberian lynx (*Lynx pardinus*). J Wildl Dis 41:810–815 16456175 10.7589/0090-3558-41.4.810

[CR25] Mehlhorn H, Schein E (1984) The piroplasm: life cycle and sexual stages. Adv Parasitol 23:37–103 6442536 10.1016/s0065-308x(08)60285-7

[CR26] Meli M, Cattori V, Martínez F, López G, Vargas A, Simón M et al (2009) Feline leukemia virus and other pathogens as important threats to the survival of the critically endangered Iberian lynx (Lynx pardinus). PLoS ONE 4:e4744 19270739 10.1371/journal.pone.0004744 PMC2649436

[CR27] Millán J, Rodriguéz A, Pérez de la Lastra J, Mangold A, de la Fuente J (2007) Prevalence of infection and 18S rRNA gene sequences of Cytauxzoon species in Iberian lynx (Lynx pardinus) in Spain. Parasitol 134:995–1001 10.1017/S003118200700248X 17326847

[CR28] Millán J, López-Bao JV, García EJ, Oleaga Á, Llaneza L, Palacios V, de la Torre A, Rodríguez A, Dubovi EJ, Esperón F (2016) Patterns of exposure of Iberian wolves (Canis lupus) to canine viruses in human-dominated landscapes. EcoHealth 13:123–134 26589403 10.1007/s10393-015-1074-8

[CR29] Millán J, Proboste T, de Mera IGF, Chirife AD, de la Fuente J, Altet L (2016) Molecular detection of vector-borne pathogens in wild and domestic carnivores and their ticks at the human–wildlife interface. Ticks Tick-Borne Dis 7(2):284–290 26643497 10.1016/j.ttbdis.2015.11.003

[CR30] Miró G, Checa R, Paparini A, Ortega N, González-Fraga JL, Gofton A, ... Irwin P (2015) *Theileria annae* (syn. *Babesia microti*-like) infection in dogs in NW Spain detected using direct and indirect diagnostic techniques: clinical report of 75 cases. Parasites & vectors, 8, 1–11. 10.1186/s13071-015-0825-2 PMC4422000 25890106

[CR31] Najm NA, Meyer-Kayser E, Hoffmann L, Herb I, Fensterer V, Pfister K et al (2014) A molecular survey of Babesia spp. and *Theileria spp*. in red foxes (*Vulpes vulpes*) and their ticks from Thuringia Germany. Ticks Tick Borne Dis 5:386–391 24717451 10.1016/j.ttbdis.2014.01.005

[CR32] Ortuño M, Nachum-Biala Y, García-Bocanegra I, Resa M, Berriatua E, Baneth G (2022) An epidemiological study in wild carnivores from Spanish Mediterranean ecosystems reveals association between Leishmania infantum, *Babesia* spp. and *Hepatozoon* spp. infection and new hosts for *Hepatozoon martis, Hepatozoon canis* and *Sarcocystis* spp. Transbound Emerg Dis 69(4):2110–2125. 10.1111/tbed.14199 10.1111/tbed.14199 34173720

[CR33] Otranto D, Cantacessi C, Pfeffer M, Dantas-Torres F, Brianti E, Deplazes P et al (2015) The role of wild canids and felids in spreading parasites to dogs and cats in Europe. Part I: Protozoa and tick-borne agents. Vet Parasitol 213:12–23 26003669 10.1016/j.vetpar.2015.04.022

[CR34] Panait LC, Mihalca AD, Modrý D, Juránková J, Ionică AM, Deak G, ... Hrazdilová K (2021) Three new species of *Cytauxzoon* in European wild felids. Veterinary Parasitology, 290, 109344 10.1016/j.vetpar.2021.109344 33465567

[CR35] Penzhorn B (2011) Why is Southern African canine babesiosis so virulent? Evol Perspect Parasit Vectors 4:51 10.1186/1756-3305-4-51 PMC3094394 21489239

[CR36] Reichard M, Van Den Bussche R, Meinkoth J, Hoover J, Kocan A (2005) A new species of Cytauxzoon from Pallas’ cats caught in Mongolia and comments on the systematics and taxonomy of piroplasmids. J Parasitol 91:420–426 15986619 10.1645/GE-384R

[CR37] Sang C, Yang Y, Dong Q, Xu B, Liu G, Hornok S, ... Hazihan W (2021) Molecular survey of *Babesia* spp. in red foxes (*Vulpes vulpes*), Asian badgers (*Meles leucurus*) and their ticks in China. Ticks and Tick-borne Diseases, 12(4), 101710 10.1016/j.ttbdis.2021.101710 33827036

[CR38] Santoro M, Auriemma C, Lucibelli MG, Borriello G, D'Alessio N, Sgroi G, ... Fusco G (2019) Molecular detection of *Babesia* spp.(Apicomplexa: Piroplasma) in free-ranging canids and mustelids from southern Italy. Frontiers in Veterinary Science, 6, 269 10.3389/fvets.2019.00269 PMC6716145 31555669

[CR39] Schnittger L, Rodriguez A, Florin-Christensen M, Morrison D (2012) *Babesia*: a world emerging. Infect Genet Evol 12:1788–1809 22871652 10.1016/j.meegid.2012.07.004

[CR40] Schnittger L, Ganzinelli S, Bhoora R, Omondi D, Nijhof AM, Florin-Christensen M (2022) The Piroplasmida *Babesia, Cytauxzoon*, and *Theileria* in farm and companion animals: species compilation, molecular phylogeny, and evolutionary insights. Parasitol Res 121:1207–1245 35098377 10.1007/s00436-022-07424-8

